# First-principles study of the electronic structures and optical and photocatalytic performances of van der Waals heterostructures of SiS, P and SiC monolayers

**DOI:** 10.1039/d0ra10808a

**Published:** 2021-04-16

**Authors:** Qaisar Alam, S. Muhammad, M. Idrees, Nguyen V. Hieu, Nguyen T. T. Binh, C. Nguyen, Bin Amin

**Affiliations:** Department of Physics, Hazara University Mansehra Pakistan; Faculty of Physics, The University of Da Nang – University of Science and Education Da Nang Vietnam; Department of Physics, Quang Binh University Quang Binh Vietnam; Institute of Research and Development, Duy Tan University Da Nang 550000 Vietnam nguyenquangcuong3@duytan.edu.vn; Faculty of Natural Sciences, Duy Tan University Da Nang 550000 Vietnam; Department of Physics, Abbottabad University of Science and Technology Abbottabad 22010 Pakistan

## Abstract

Designing van der Waals (vdW) heterostructures of two-dimensional materials is an efficient way to realize amazing properties as well as open up opportunities for applications in solar energy conversion, nanoelectronic and optoelectronic devices. The electronic structures and optical and photocatalytic properties of SiS, P and SiC van der Waals (vdW) heterostructures are investigated by (hybrid) first-principles calculations. Both binding energy and thermal stability spectra calculations confirm the stability of these heterostructures. Similar to the corresponding parent monolayers, SiS–P (SiS–SiC) vdW heterostructures are found to be indirect type-II bandgap semiconductors. Furthermore, absorption spectra are calculated to understand the optical behavior of these systems, where the lowest energy transitions lie in the visible region. The valence and conduction band edges straddle the standard redox potentials of SiS, P and SiC vdW heterostructures, making them promising candidates for water splitting in acidic solution.

## Abstract 2

The electronic structures and optical and photocatalytic properties of SiS, P and SiC van der Waals (vdW) heterostructures are investigated by (hybrid) first-principles calculations.

## Graphical abstract

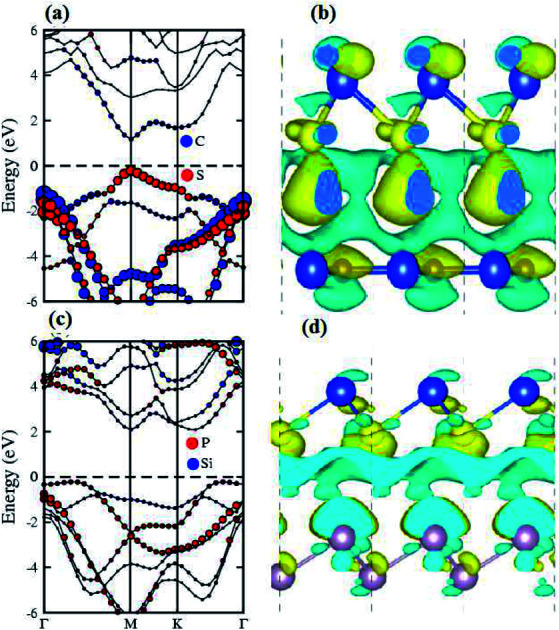

## 1 Introduction

After the discovery of graphene,^1^ two dimensional (2D) materials, such as hexagonal boron nitride (h-BN),^2^ transition metal dichalcogenides (TMDCs),^3^ silicon carbide (SiC),^4^ MXenes,^5^ phosphorene^6^ and BSe,^7^ have shown superior performances over their bulk counterparts because of their large surface areas and high concentrations of open-transport channels.^8,9^ Among these 2D materials, much attention has been paid to the silicon sulfide (SiS) monolayer, which is a group IV–VI material.^10^ This 2D material consists of three structure types, the α-SiS, β-SiS and *Pma*2-SiS isomers.^12–14^ Among these types, Yang *et al.*^14^ demonstrated theoretically that the *Pma*2 type is more energetically stable than α-SiS and β-SiS. Jing *et al.*^15^ predicted that the SiS monolayer exhibits a large negative Poisson’s ratio, tunable electronic properties under strain and anisotropic carrier mobility. Thus, this material is a potential candidate for optoelectronic applications. Recently, the graphene-like 2D SiC monolayer has been predicted to be structurally stable and exhibits promising applications.^16^ It is obvious that the SiC monolayer is a nonmagnetic semiconductor and it has a planar atomic structure. In addition, the large in-plane stiffness, strong thermostability and high carrier mobility make the SiC monolayer a promising candidate for designing next-generation electronic devices.^17,18^ More recently, Zhu *et al.*^19^ predicted that quasi-2D blue phosphorene (P) can be exfoliated from the bulk layer of blue phosphorus. They also confirmed that the single-layer P is thermally and dynamically stable at room temperature.

Currently, the stacking of 2D layers *via* van der Waals (vdW) interactions in the form of a heterostructure^20,21^ is a constructive tool to design viable electronic products.^22,23^ In vdW heterostructures, there is no direct chemical bonding between the constituent layers, therefore the condition of lattice mismatch can be solved by varying the lattice constants. Furthermore, one can easily control the combination of the photogenerated-carriers with type-II band alignment and tune the properties of the corresponding 2D materials.^24–26^ Recently, the physical properties of GeC–MSSe (M = Mo, W),^27^ ZnO–MSSe (M = Mo, W),^28^ g-GaN, blue phosphorene, SiC, ZnO,^29^ BSe–SiC–ZnO,^30^ MoXY–WXY ((X ≠ Y) = S, Se, Te)^31^ and MoSSe–WSSe^32^ vdW heterostructures have been investigated. The electronic and photocatalytic properties of SiC-based, SiS-based and P-based vdW heterostructures have also been investigated recently.^33–36^ However, the combination of SiC(P) and SiS monolayers has not yet been investigated thoroughly.

Furthermore, it is clear that SiC and P based heterostructures exhibit suitable band edge positions close to to the redox potentials of water, which makes them potential photocatalysts for water splitting. It is natural to check whether SiC, P and SiS monolayers can form stable vdW heterostructures or not. To answer this question, we have systematically investigated the electronic structures, band edge alignments and optical properties of the combination between SiC(P) and SiS to form SiS–SiC (SiS–P) vdW heterostructures using density functional theory. The photocatalytic responses of these vdW heterostructures are also investigated. Our calculations show that SiS–SiC and SiS–P vdW heterostructures could be promising candidates for visible light photocatalysis and optoelectronic devices.

## 2 Computational methods and models

In this paper, comprehensive insight is gained into the physical properties of the SiS, P and SiC monolayers and their vdW heterostructures. The thermal stabilities of the heterostructures and corresponding monolayers are investigated by *ab initio* molecular dynamics (AIMD) simulations^37^ with an 8 × 8 × 1 supercell through the Nose thermostat algorithm at a temperature of 300 K for a total of 6 ps with a time interval of 1 fs. Density functional theory (DFT)^38^ calculations with Grimme’s (DFT-D2) empirical dispersion correction^39^ in the Vienna *ab initio* simulation package^40–42^ are used to investigate the electronic structures and optical and photocatalytic performances of the SiS, P and SiC van der Waals heterostructures. We fixed a plane-wave cut-off of 500 eV, *Γ*-centered Monkhorst–Pack *k*-meshes of 6 × 6 × 1 (12 × 12 × 1) for the structural relaxation (optimized structures) and a vacuum layer of 25 Å along the *z*-axis in these calculations. The geometric relaxations are performed by the Perdew–Burke–Ernzerhof (PBE) functional,^43^ until the forces converged to 10^−4^ eV Å^−1^ and the energy to 10^−5^ eV, and the HSE06 (Heyd–Scuseria–Ernzerhof) functional^44^ is used for electronic structure calculations. Furthermore, the Bethe–Salpeter equation (BSE) is solved using the GW_0_ method to explore the optical spectra in terms of the imaginary part of the dielectric function of the hBP–XMY (M = Mo, W; (X ≠ Y) = S, Se, Te) vdW heterostructures.^45,46^ Furthermore, the photocatalytic responses of the above studied materials are investigated by using Mulliken electronegativity.

## 3 Results and discussion

**Fig. 1:**
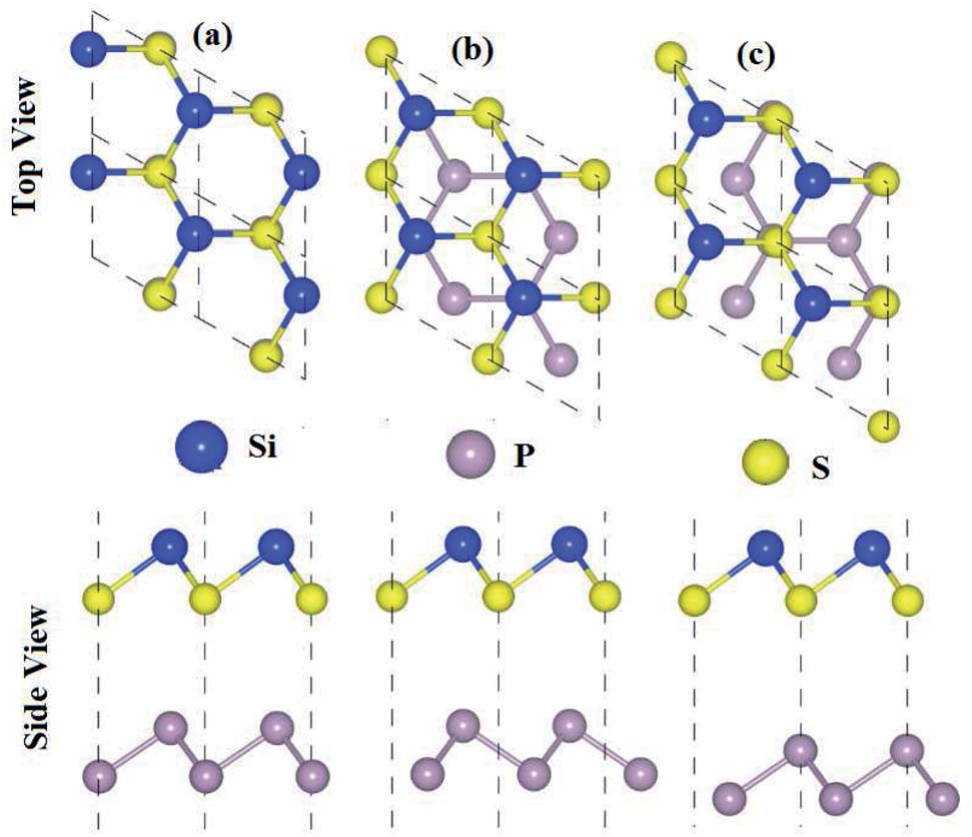
High symmetry stacking modes of the SiS–P heterostructures for different stacking configurations: (a) i-stacking, (b) ii-stacking, (c) iii-stacking, (d) iv-stacking, (e) v-stacking and (f) vi-stacking. The unit cells are marked by dashed lines.

The optimized lattice constants, bond lengths, band structures and bandgap values of the pristine SiS, P and SiC monolayers presented in Table 1 and Fig. 1 are in good agreement with the previous available findings.^10,30,47^ The small lattice mismatches (<2%) between these monolayers allow for the fabrication of their vdW heterostructures. Controlling the orientations of the monolayers during mechanical exfoliation and/or the subsequent stacking order in the fabrication of vdW heterostructures is quite difficult. Also, the interfacial properties are sensitive to the specific contacted atoms and local configurations. Therefore, using optimized lattice constants, three (six) possible stacking configurations of the SiS–P (SiS–SiC) vdW heterostructures are investigated (see Fig. 1).

**Table 1:** Lattice constants (*a*), bond lengths (M–X), band gaps (*E*_g-HSE_, *E*_g-GW_0__), work functions (*ϕ*), binding energies (*E*_i_, *E*_ii_, *E*_iii_, *E*_iv_, *E*_v_, *E*_vi_) and interlayer distances (*d*) for monolayer and model-I(-II) vdW heterostructures

Janus monolayer	SiS	P	SiC	SiS–P	SiS–SiC
*E*_i_ (eV)	—	—	—	−0.365	−0.484
*d*_i_ (Å)	—	—	—	3.31	3.19
*E*_ii_ (eV)	—	—	—	−0.362	−0.433
*d*_ii_ (Å)	—	—	—	3.32	3.66
*E*_iii_ (eV)	—	—	—	−0.335	−0.483
*d*_iii_ (Å)	—	—	—	3.87	3.21
*E*_iv_ (eV)	—	—	—	—	−0.472
*d*_iv_ (Å)	—	—	—	—	3.27
*E*_v_ (eV)	—	—	—	—	−0.475
*d*_v_ (Å)	—	—	—	—	3.26
*E*_vi_ (eV)	—	—	—	—	−0.468
*d*_vi_ (Å)	—	—	—	—	3.36
*a* (Å)	3.31	3.27	3.09	3.299	3.099
X–Y (Å)	2.10	2.26	2.10	2.279	2.287
Si–P/Si–C (Å)	—	—	—	2.223	1.847
*E*_g-PBE_ (eV)	2.17	2.16	2.52	1.410	0.70
*E*_g-HSE_ (eV)	3.01	2.88	3.39	2.220	2.210
*ϕ* (eV)	—	—	—	1.24	1.20

**Fig. 2:**
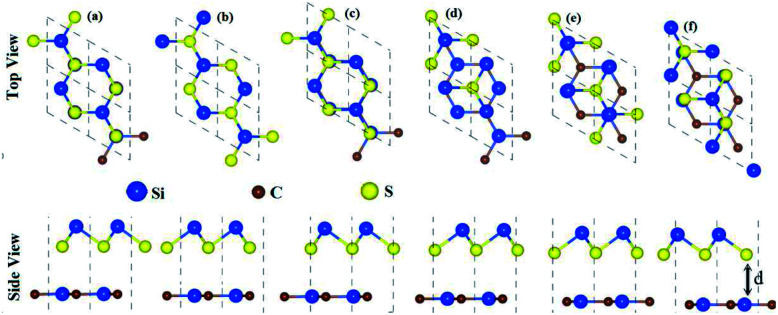
High symmetry stacking modes of the SiS–SiC heterostructures for different stacking configurations: (a) i-stacking, (b) ii-stacking, (c) iii-stacking, (d) iv-stacking, (e) v-stacking and (f) vi-stacking. The unit cells are marked by dashed lines.

**Fig. 3:**
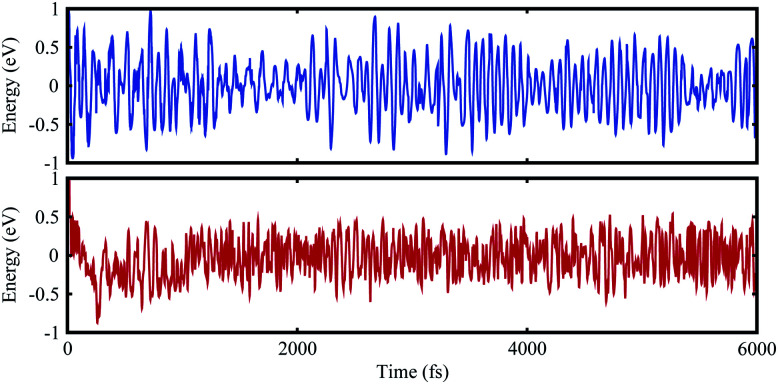
*Ab initio* molecular dynamics calculation of the thermal stability of the SiS–P (blue) and SiS–SiC (red) heterostructures.

In the case of the SiS–P heterostructure, the stacking orders are: (i) the P atoms are located on top of both the Si and S atoms, (ii) the P atoms are located on the top of the Si atoms and the hexagonal centers, (iii) the P atoms are placed on top of the S atoms and the hexagonal centers, as shown in Fig. 1. In the case of the SiS–SiC heterostructure, the six possible stackings are: (i) the Si (C) atoms are on top of the Si (S) atoms, (ii) the Si (S) atoms are on top of C (Si), (iii) the Si atoms are on top of the hexagonal centers, while the C atoms are on top of the Si atoms, (iv) the Si atoms are on the hexagonal centers and the C atoms are on top of the S atoms, (v) the C atoms are on the hexagonal center, while the Si atoms are on top of the Si atoms, (vi) the C atoms are on the hexagonal center, while Si is on top of the Si atoms, as shown in Fig. 2. The binding energy (*E*_b_) is the difference between the total energy of the heterostructures and the corresponding parent monolayers, calculated by: *E*_b_ = *E*_heterostructure_ − *E*_monolayer-i_ − *E*_monolayer-ii_. The binding energies and the interlayer distances, presented in Table 1, show that stacking-i is the most favorable stacking due to the smallest *E*_b_ and shortest interlayer distance. Furthermore, the *ab initio* molecular dynamics calculations for the thermal stability, presented in Fig. 3, show that there is no geometric reconstruction or bonds broken after heating the system at 300 K for 6 ps, hence these systems are also stable even at room temperature.

**Fig. 4:**
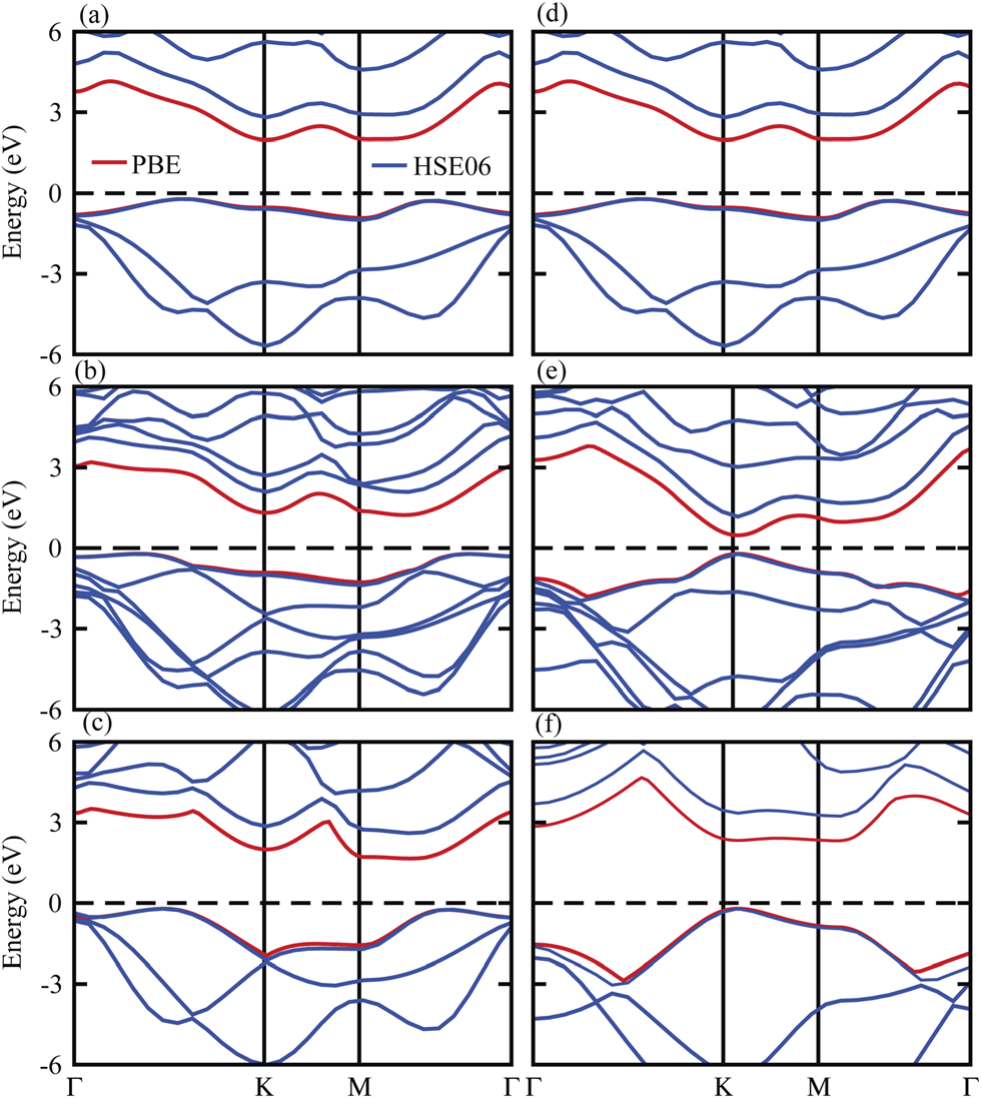
Band structures of (a) SiS, (b) SiS–P, (c) P, (d) SiS, (e) SiS–SiC and (f) SiC with the PBE (red) and HSE06 (blue) functionals.

The band structures in Fig. 4 show that the SiS and P monolayers are indirect band gap semiconductors, while SiC is a direct band gap semiconductor. Interestingly, the SiS–SiC (SiS–P) vdW heterostructure in stacking-i is a direct (indirect) band gap semiconductor with the VBM at the *K* (*Γ*–*K*)-point, while the CBM is at the *M*-point of the Brillouin zone. Using both the PBE and HSE06 functionals, the band gap values presented in Table 1 show that this stacking reduces the band gap with respect to the corresponding monolayers and this is in good agreement with previous reports.^10,30,47^

**Fig. 5:**
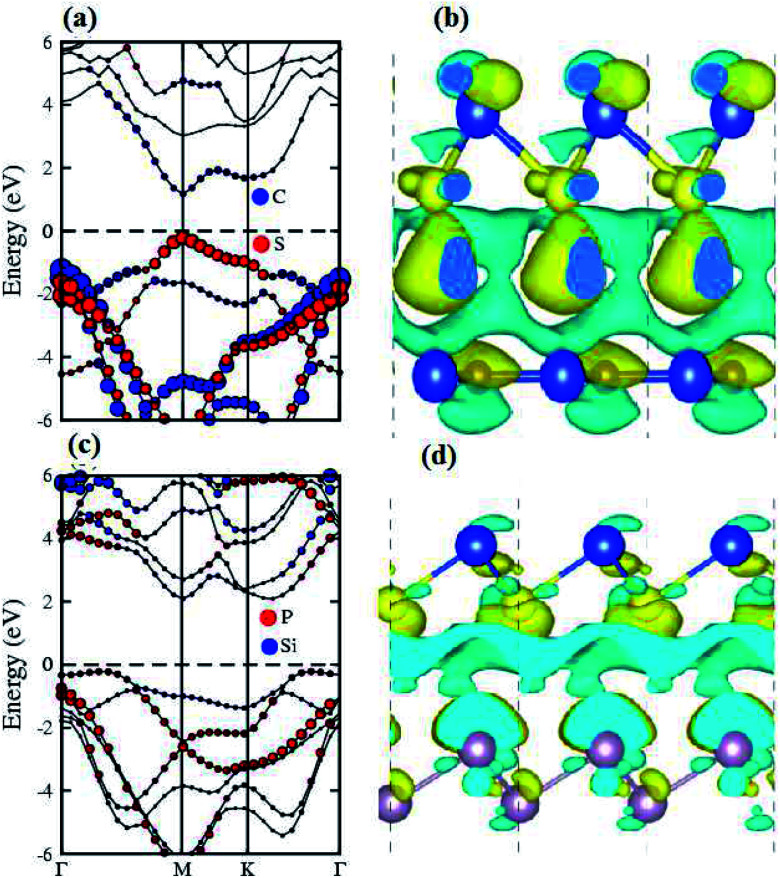
Weighted band structures (a and c) and Bader charge analysis (b and d) of the SiS–SiC (a and b) and SiS–P (c and d) heterostructures with PBE and HSE06.

**Fig. 6:**
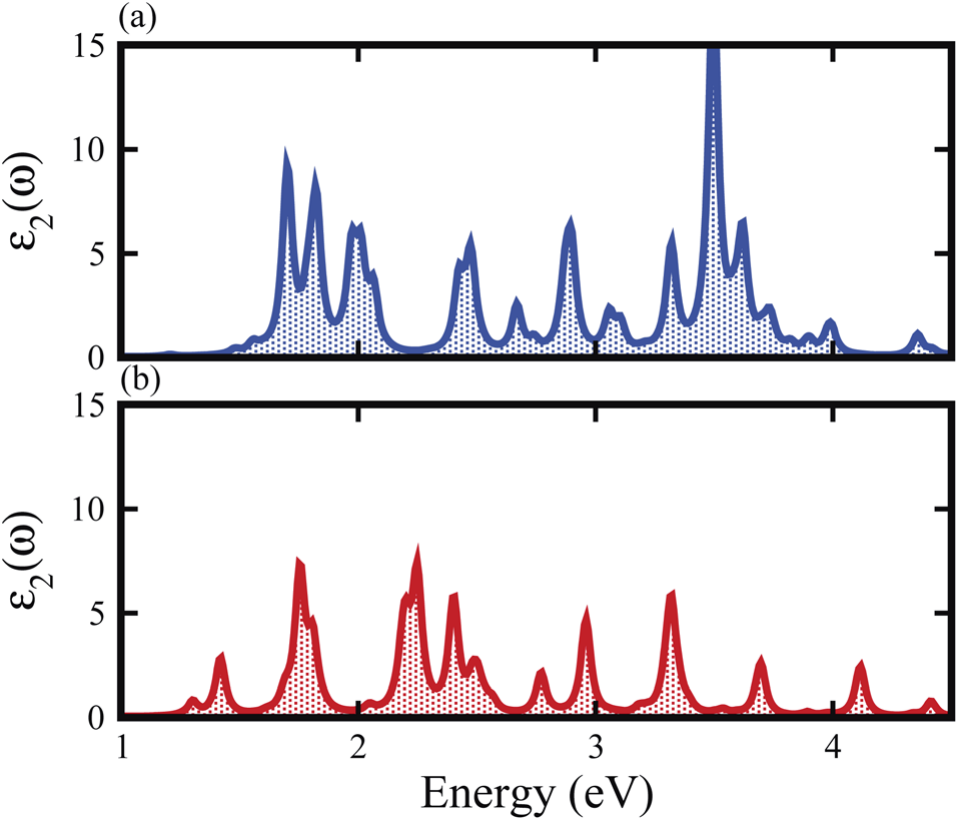
Imaginary part of the dielectric function of the (a) SiS–P and (b) SiS–SiC heterostructures.

The contributions of the atomic sites in the VBM and CBM are further elaborated by the weighted band structures presented in Fig. 5. In the case of the SiS–SiC vdW heterostructure, the VBM is dominated by the p_*z*_ orbital of S in the SiS layer at the *M*-point of the BZ, whereas the CBM is due to the p_*z*_ orbital of C in the SiC layer at the *M*-point of BZ, hence this heterostructure shows type-II band alignment, as shown in Fig. 5(a). Similar to the SiS–SiC vdW heterostructure, SiS–P presents type-II band alignment where the VBM and CBM are located on the *Γ*–*K* and *K*-points of the BZ, respectively, as shown in Fig. 5(c). Type-II band alignment spontaneously separates the electron–hole pairs, enabling high efficiency optoelectronics and solar energy conversion.^47^ The variation of the band structure of the vdW heterostructures with respect to the corresponding monolayers is due to the transfer of charges from one monolayer to the other. Therefore, we also analyse the Bader population and calculate the charge density difference by *ρ* = *ρ*_heterostructure_ − *ρ*_monolayer-i_ − *ρ*_monolayer-ii_, where *ρ*_heterostructure_ is the charge density of the vdW heterostructure and *ρ*_monolayer-i_ and *ρ*_monolayer-ii_ are the charge densities of the corresponding isolated monolayers, as shown in Fig. 5. In the case of the SiS–SiC vdW heterostructure, charges are transferred from the SiS monolayer to the SiC monolayer due to the Si atoms, while in the SiS–P heterostructure the charges are transferred from P to the SiS monolayer due to the P atoms. One can see that the SiS (P) layer donates electrons to the SiC (SiS) layer, which leads to p-doping in SiS (P) and n-doping in SiC (SiS) in the SiS–SiC (SiS–P) vdW heterostructures, as shown in Fig. 5. It is also clear from the figure that the localized layers in the CBM and VBM (discussed above) potentially act as the electron donors and the electron acceptors, respectively, in the corresponding heterostructures. The separation of the photo-generated charge carriers indicates that these vdW heterostructures have potential applications in solar energy conversion.^48,49^ In addition, it can be seen that the band gaps of the SiS–P and SiS–SiC heterostructures are calculated to be 2.22 eV and 2.21 eV, respectively, which are in the energy range of 1.5–3.0 eV.^11^ These band gap values demonstrate that they could absorb more abundant visible light with high solar energy conversion efficiency. The imaginary part of the dielectric function *ε*_2_(*ω*) is depicted in Fig. 6. It is obvious that the exciton peaks are observed at 1.63 eV and 1.45 eV for the SiS–P and the SiS–SiC heterostructures, respectively. These values indicate strong modifications in the positions of excitons in the heterostructures, suggesting that this is promising for controlling the exciton–phonon interactions or coupling at the nanoscale.

**Fig. 7:**
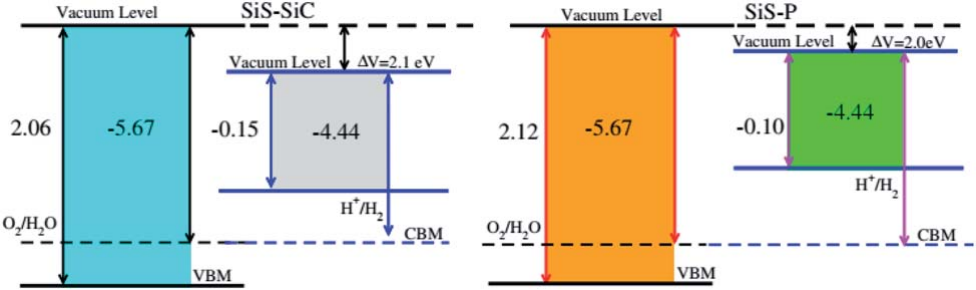
Valence and conduction band edge potentials against the vacuum potentials of the SiS–P and SiS–SiC heterostructures.

Photocatalytic water splitting is an attractive technology for producing clean and renewable energy without pollution by using semiconductors as hosts for the conversion of water to hydrogen using solar light.^50–52^ Therefore, it is possible to find a suitable photocatalyst, which can efficiently utilize solar energy to dissociate water and produce hydrogen.^53–55^ In this process, the redox potential of H^+^/H_2_ (0 eV) has to be less negative than the bottom level of the conduction band and the redox potential of O_2_/H_2_O (1.23 eV) has to be less positive than the top level of the valence band.^56^ This implies that the band gap of the considered heterostructure must be greater than 1.23 eV. The calculated band gaps of both the SiS–SiC and SiS–P vdW heterostructures are greater than 1.23 eV. Therefore, these materials must show good abilities for water splitting at pH = 0. In addition, the photocatalytic water splitting performance of the SiS–SiC and SiS–P vdW heterostructures is investigated using Mulliken electronegativity using *E*_VBM_ = *χ* − *E*_elec_ + 0.5*E*_g_ and *E*_CBM_ = *E*_VBM_ − *E*_g_ at pH = 0,^57,58^ where *χ* is the geometric mean of the Mulliken electronegativities and *E*_g_ is the calculated band gap value. Fixing the Fermi level at −4.44 eV, the band edge potentials of the SiS–SiC and SiS–P heterostructures in connection with the water reduction and oxidation potential levels are displayed in Fig. 7. We set the VB/CB at 1.23 eV/0 eV which is equal to −5.73 eV/−4.50 eV for an aqueous solution at pH = 0. It is clear that both the VBM and the CBM edges in SiS–SiC and SiS–P straddle the standard redox band edges and enhance the ability to perform both reduction and oxidation, hence making them potential candidates for water splitting in acidic solution. Depending on the appropriate electronic band structure for overall water splitting with proper valence and conduction band edge alignment, these heterostructures can be considered as highly efficient photocatalysts for the conversion of water to hydrogen using solar light.^59,60^

## 4 Conclusion

First-principles (hybrid) calculations are performed to investigate the structural, electronic and optical properties and the photocatalytic performances of SiS, P and SiC vdW heterostructures. The thermal stability confirms the dynamic stability up to room temperature of the most favorable stacking of the corresponding vdW heterostructures. All of the heterostructures have an indirect bandgap nature with type-II band alignment. The calculated electron carrier mobility is higher than the hole carrier mobility, suggesting that these heterostructures can be utilized for hole/electron separation. Also, absorption spectra are calculated to understand the optical behavior of these systems, where the lowest energy transitions lie in the visible region. More interestingly, all of these heterostructures have the capability to perform both reduction and oxidation, revealing them to be potential candidates for promising applications in photocatalytic water splitting.

## Conflicts of interest

There are no conflicts to declare.

## Supplementary Material
